# Association of Parental Feeding Styles with Body Composition Among Children in Two Regions in China

**DOI:** 10.3390/nu17132197

**Published:** 2025-06-30

**Authors:** Chao Li, Sha Liu, Dingkang Wang, Mengzi Sun, Jie You, Bizhong Che, Wen Zhang, Wei Wei, Yaling Zhao, Youfa Wang

**Affiliations:** 1 Global Health Institute, School of Public Health, Xi’an Jiaotong University, Xi’an 710061, China; lc18182582951@163.com (C.L.); liusha09152022@163.com (S.L.); dkwang@xjtu.edu.cn (D.W.); sunmengzi@xjtu.edu.cn (M.S.); boche@xjtu.edu.cn (B.C.); 2 Ministry of Science and Education, Nuclear Industry 215 Hospital of Shaanxi Province, Xianyang 712000, China; 3 Luohu District Center for Disease Control and Prevention, Shenzhen 518001, China; youjie-45@126.com (J.Y.); wen--100@163.com (W.Z.); 4 Institute of Health Sciences, Chinese Medical University, Shenyang 110122, China; wei378023020@gmail.com

**Keywords:** parental feeding, child, body composition, obesity, regional differences

## Abstract

**Background:** This study aimed to investigate the correlation between parental feeding practices and children’s body composition in two Chinese regions with distinct socioeconomic characteristics. **Methods:** A cross-sectional study was conducted in Shenzhen (economically developed) and Yulin (economically underdeveloped) regions. Data were collected in 2023 from 1298 (age 8–10 years) children and their parents in two regions. Overweight/obesity was defined by Chinese national standards (WS/T 586-2018), and parental feeding practices were assessed using a 26-item child feeding questionnaire (CFQ). Multivariable regression analysis was performed to assess whether the association between parental feeding practices and obesity in children differs by regions. **Results:** A total of 1298 participants were included, with 678 male students (52.23%) and a mean age of 10.65 ± 0.86 years. In two regions, children with higher pressure to eat (PE) scores had lower rates of overweight, obesity and central obesity. Significant positive associations were observed between children’s body composition and parental feeding practices, including PE, perceived child weight (PCW), and concern about child weight (CN) (all *p* < 0.001). In regional interaction analysis, PCW had significant positive associations with fat mass index (FMI) (β = 0.32, 95% CI = 0.18, 0.46). Meanwhile, CN also had significant positive associations with both FMI (β = 0.44, 95% CI = 0.34, 0.54) and fat-free mass index (FFMI) (β = 0.07, 95% CI = 0.02, 0.11) (all *p* < 0.001). **Conclusions:** Obesity, central obesity, and body composition in children were associated with parental feeding practices in the two regions. CN was associated with higher risk of obesity and central obesity in the two regions. Future efforts to prevent obesity in children may optimize parental feeding practices, especially more scientific awareness of children’s weight status while reducing undue concern.

## 1. Introduction

Children’s nutritional health has emerged as a critical global public health concern, with the prevalence of obesity among children escalating over recent decades [1,2]. According to an article published in *The Lancet*, since 1975, the global age-standardized obesity prevalence among children and adolescents aged 5–19 has surged dramatically—from just 0.7% to 5.6% in girls and from 0.9% to 7.8% in boys by 2016 [3]. A report by the World Obesity Federation estimates that by 2035, approximately 383 million children and adolescents aged 5–19 will be overweight or obese, globally [4]. The ramifications of obesity in children are profound and multifaceted, including persistent obesity into adulthood, heightened risk for various non-communicable diseases, like type 2 diabetes, cardiovascular disease, chronic kidney disease, and cancer, and increased mortality along with recognized complications related to obesity [5,6].

Body mass index (BMI) is widely utilized in clinical settings to assess health status and screening for overweight and obesity, as it accounts for height and exhibits a general correlation with body fat content [7]. However, BMI has limitations, particularly in distinguishing between fat mass and fat-free mass. Recent studies have emphasized that body composition analysis provides a more precise evaluation of body fat content and distribution [8,9], making it crucial for screening obesity and preventing related complications and diseases [10]. Key body composition indices—such as body fat mass (BFM), body fat percentage (PBF), fat-free mass (FFM), and fat-free mass index (FFMI)—have demonstrated strong associations with obesity in children, thus providing predictive insights for obesity-related chronic conditions, such as hypertension, type 2 diabetes, dyslipidemia, and fatty liver disease [11]. Notably, parental feeding practices play a pivotal role in shaping children’s eating behaviors and, consequently, their weight status. These practices—encompassing attitudes, behaviors, and emotional responses toward feeding—can have lasting effects on a child’s relationship with food and long-term health outcomes [12,13,14]. A Portuguese cohort study found a bidirectional relationship between parental feeding practices and children’s BMI [15]. A longitudinal study in a Chinese mega-city found that maternal and paternal education levels moderated the relationship between eating pressure and young children’s weight status [16]. Furthermore, the prevalence of overweight and obesity among children was also linked to parental feeding practices [17]. On a broader scale, socioeconomic factors also contribute significantly to shaping these patterns. Economic disparities between regions influence access to resources like education, healthcare, and social support systems, which in turn affect parenting styles and, ultimately, children’s nutritional outcomes. These interconnected variables demonstrate how crucial it is to approach the prevention of obesity in children from a holistic, multi-faceted perspective, addressing both behavioral and structural determinants [18].

This study examined sex- and region-specific variations in key factors influencing parental feeding practices and children’s body composition across two distinct regions in China. Additionally, it explored the relationship between parental feeding practices and children’s body composition outcomes. This study provides a more accurate assessment of obesity by distinguishing body composition indicators (such as FMI and FFMI) rather than BMI alone, and for the first time in a cross-regional analysis to investigate the relationship between parental feeding practices and body composition and obesity-related indicators among children with obesity. Through this cross-sectional study, we hypothesized that parents’ concern about child weight (CN) and perceived child weight (PCW) were positively correlated with children’s body composition (FMI and FFMI) and risk of obesity, and pressure to eat (PE) was associated with a lower risk of obesity and central obesity, and these associations were affected by region.

## 2. Materials and Methods

### 2.1. Study Design and Participants

This cross-sectional study utilized data from two distinct initiatives: the “Shenzhen Children’s Health Development Program” in Shenzhen, representing China’s economically developed southeastern coastal region, and the “Survey and Improvement of Nutritional Status of School-age Children in Poverty-stricken Areas of China” project in Yulin, representing the economically underdeveloped northwestern region. A cluster random sampling method was employed, wherein entire classes were randomly selected, and all students within the selected classes were surveyed. Between October and December 2023, 1393 third-grade students from eight elementary schools in Luohu District, Shenzhen, participated in the survey, with body composition data successfully collected from 955 students. Meanwhile, in Yulin, 995 students from four elementary schools in Zizhou and Qingjian Counties were surveyed, with body composition data successfully collected from 692 students. The total study population consisted of 2388 students and their parents from the 2023 academic year. After excluding participants with missing basic information or key variables, a total of 1298 valid samples (comprising parents and children) were retained for subsequent analysis. Based on the actual sample size, the empirical value of Power = 0.89 was obtained, which was higher than the recommended minimum value of 0.80, indicating that the study sample size was reasonable.

### 2.2. Questionnaire Data Collection

Parents completed the questionnaire survey. The students completed their questionnaires independently. The parent questionnaire was an online survey completed by the student’s mother or primary guardian. The parent questionnaire used Child Feeding Questionnaire (CFQ) adapted 26 items about the family’s general situation, parents’ health, food preferences of the student and the family, and parental feeding experiences. The CFQ has demonstrated good validity and reliability among Chinese children [19] and adolescents [20].

### 2.3. Anthropometric and Body Composition Measurements

All study participants underwent standardized anthropometric assessments conducted by trained investigators, including measurements of height, weight, waist circumference, and body composition indices. Height and weight were measured using the HGM-700 scale, with students barefoot and wearing light clothing, and readings taken once stable. Waist circumference was measured with an inelastic tape, requiring students to stand upright and lift their shirts to expose the waist, with measurements accurate to 0.1 cm. Body composition was assessed using the TANITA MC-780MA body composition analyzer, following the manufacturer’s guidelines [21].

### 2.4. Study Variables

The diagnostic criteria and definitions for the key health variables fall into three categories: The outcome variables included obesity-related and body composition indices, exposure variables were parental feeding practices, and covariates included primarily demographic characteristics and other relevant factors. Children’s BMI was calculated using the standard formula: BMI = weight (kg)/height (m)^2^. The Chinese Obesity Working Group was used to classify the criteria for overweight and obesity screening in Chinese school-aged children and adolescents. According to the “WS/T 586-2018: Screening for Overweight and Obesity among School-aged Children and Adolescents” guidelines. Overweight and obesity are defined based on age- and sex-specific BMI percentiles: underweight/normal weight (<85th percentile), overweight (≥85th percentile and <95th percentile), and obesity (≥95th percentile). Waist-to-height ratio (WHtR) = waist circumference (cm)/height (cm). Principal component analysis was employed to identify FMI and FFMI as key indicators of body composition for this study. FMI was calculated as BFM divided by height squared (kg/m^2^), and FFMI was calculated as FFM divided by height squared (kg/m^2^). BIA-derived measures (e.g., FMI) rely partially on weight-based equations, potentially limiting precision.

Children’s primary caregivers rated their agreement with questionnaire items using a 5-point Likert scale. The Parental Feeding Practices Scale comprises seven dimensions, namely: Monitoring (MN), Pressure to Eat (PE), Restriction (RST), Perceived Child Weight (PCW), Concern About Child Weight (CN), Perceived Parent Weight (PPW), and Food as Reward (FR). The overall Cronbach’s alpha coefficient across the seven subscales was 0.843. The Cronbach’s alpha coefficients of the two regions were 0.875 and 0.794. The Cronbach’s alpha coefficients for MN, PE, RST, PCW, CN, PPW, and FR were 0.933, 0.677, 0.877, 0.796, 0.817, 0.822, and 0.818, respectively Covariates were selected based on previous literature review and data from both children and parents. Given that demographic characteristics and behavioral factors can influence children’s body composition, these covariates were controlled for in subsequent analyses. Controlled covariates in this study included: age (in years), gender, city of residence (Yulin or Shenzhen), parents’ education level, occupation, monthly family income, parental body mass index, children’s diet, and physical activity [22].

### 2.5. Statistical Analysis

Anthropometric data from the “Survey on Nutritional Status and Improvement of School-age Children in Poverty-stricken Areas of China” and the “Shenzhen Children’s Health Development Project” databases were double-entered and organized using Microsoft Excel 2019 to ensure data accuracy. Questionnaire data were double-entered and calibrated using EpiData version 3.1 software. Statistical analyses were performed using STATA 18.0 (Stata Corp, College Station, TX, USA), with statistical significance set at two-sided *p* < 0.05. Descriptive statistics were presented as frequencies and percentages for categorical variables, and means with standard deviations for continuous variables. Analysis of variance (ANOVA) was employed to assess baseline differences between child and parent characteristics. The two independent sample *t*-tests and chi-square tests were used for group comparisons, respectively, between different regions to describe the basic characteristics. One-way analysis of variance (ANOVA) was conducted to examine baseline differences in child and parental characteristics across tertiles of parental feeding practices. Factor analysis was used to extract representative indicators of body composition. The Kaiser–Meyer–Olkin (KMO) measure and Bartlett’s test of sphericity were used to assess suitability for factor analysis (with KMO > 0.7 and *p* < 0.05 indicating appropriateness). After applying orthogonal rotation, variables with absolute factor loadings greater than 0.7 were selected as representative indicators. Pearson’s correlations using Graphpad Prism 9.2 generate correlation heatmap. Multivariable regression analysis was applied to analyze associations among outcome variables and parental feeding practices [23]. Considering the potential autocorrelation among the seven dimensions of parental feeding practices, a stricter significance threshold (*p* < 0.01) was applied for these main hypotheses in the multivariable regression analysis. Other analysis statistical significance was set at a two-sided *p*-value of less than 0.05.

## 3. Results

### 3.1. Characteristics of the Study Population

Participant characteristics are shown in Table 1. A total of 1298 students were investigated, mean age of 10.65 ± 0.86 years and 678 male students (52.23%). The overall prevalence of overweight and obesity among children was 34.52%, with rates of 37.83% in Yulin and 31.69% in Luohu District, Shenzhen,. and there were regional differences (*p* < 0.05). In Yulin, 68.31% of mothers were unemployed, and over 70% of parents had middle school or lower education. In contrast, in Shenzhen, 48.75% of mothers worked as employees or staff and there were over 50% of parents had college and above education, reversely. Monthly family income in Yulin was primarily under 10,000 yuan (94.46%), while in Shenzhen was mostly over 10,000 yuan (78.80%), indicating contrasting socioeconomic statuses between the two regions. Except for PE and FR (*p* > 0.05), significant differences were found for all of the remaining five dimensions (all *p* < 0.001), as shown in Table 1.

### 3.2. Characteristics of Parental Feeding Practices and Body Composition

The study revealed that higher scores in PCW, concern about CN, and PPW were significantly associated with increased prevalence of overweight and obesity. Children with PE scores had lower rates of overweight(including obesity) and central obesity (Yulin: Low 38.00% vs. High 21.37%; Low 38.48% vs. High 21.37; Shenzhen: Low 42.23% vs. High 31.10%; Low 31.88% vs. High 21.55%; a *p* < 0.001). In contrast, higher PCW, CN, and PPW scores were associated with higher rates of overweight, obesity, and central obesity. Compared with Shenzhen, children in Yulin with higher FR scores exhibited significantly lower rates of overweight, obesity, and central obesity (all *p* < 0.05). Additionally, about children’s body composition, higher PCW, CN, and PPW scores were associated with increased FMI and FFMI (all *p* < 0.001), while higher PE scores were associated with lower FMI and FFMI (all *p* < 0.05) in both regions, These trends were consistent with patterns observed in children’s weight status (Table 2).

### 3.3. Multivariate Analysis Between Parental Feeding Practices and Body Composition

**Figure 1:**
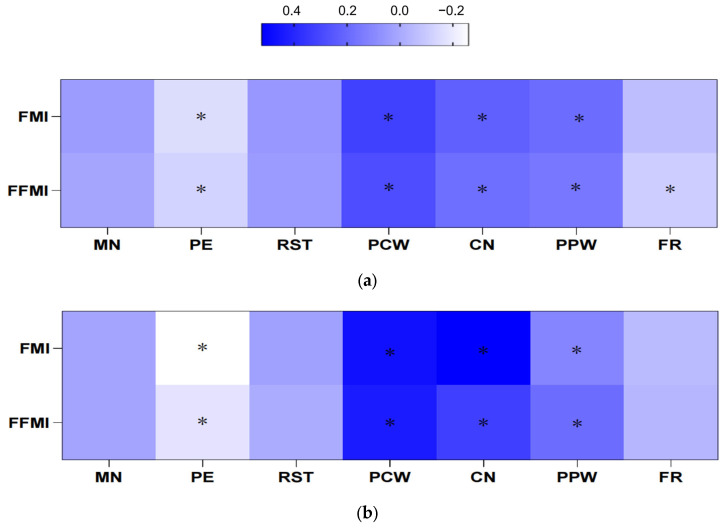
Pearson’s correlations between CFQ factors and body composition in two regions. Abbreviations: FMI: Fat mass index; FFMI: Fat free mass index. MN: Monitoring; PE: Pressure to eat; RST: Restriction; PCW: Percived child weight; CN: Concern about child weight; PPW: Percived parent weight; FR: Food as reward. *: *p* < 0.05. (**a**) Yulin, (**b**) Shenzhen.

Figure 1 illustrates the correlation between parental feeding practices and children’s body composition indicators. Significant positive correlations were observed among PCW, CN and PPW with both FMI and FFMI. Conversely, PE was significantly negatively correlated with both FMI and FFMI. In Yulin, FR was significantly negatively correlated with FFMI (*p* < 0.05). In contrast, no significant associations between FR and body composition indicators were observed in Shenzhen. In Yulin, the strongest correlations were found on PCW and CN patterns (both *p* < 0.001). Significant positive correlations were also observed between PCW, CN and PPW with body composition indicators indicators among children in Shenzhen. However, no significant correlations were found in MN and RST patterns. Positive associations were particularly noted between CN and FMI (*r* = 0.521). Similarly, a significant negative association between PE and children’s body composition was observed, with this correlation being more pronounced in Shenzhen compared to Yulin.

### 3.4. Analysis of Factors Influencing Children’s Body Composition

In Yulin, the negative association between PE and FMI was more pronounced, with an interaction coefficient of β = −0.14 (*p* < 0.001). PCW had significant positive associations with FMI (β = 0.32, 95% CI = 0.18, 0.46). Meanwhile, CN also had significant positive associations with both FMI (β = 0.44, 95% CI = 0.34, 0.54) and FFMI (β = 0.07, 95% CI = 0.02, 0.11) in regional interaction analyses (*p* < 0.001), as shown in Table 3. In two regions, multivariable regression analysis revealed that PE was a statistically significant negative predictor for both FMI and FFMI. Conversely, PCW, CN, and PPW were significant positive predictors for both FMI and FFMI in two regions. PPW had a significant positive association with FMI (*β* = 0.25, 95% CI = 0.13, 0.27; *p* < 0.001) in Yulin Similarly, PE was also a statistically significant negative predictor of all body composition indicators, while PCW and CN were stronger positive predictors of higher body composition. Moreover, the association between PCW and FMI was stronger (Yulin *β* = 0.32 vs. Shenzhen *β* = 0.15, *P*_interaction_ < 0.001). PPW had a significant positive association with FFMI (*β* = 0.12, 95% CI = 0.07, 0.17; *p* < 0.001) in Shenzhen. Furthermore, FR was a statistically significant negative predictor for FFM and FFMI in Yulin; while there had no statistical significance was observed for FR (all *p* > 0.01) when compared between the two regions, as shown in Table 3.

Parental feeding practices were associated with children’s weight status, as detailed in Table 4. PE functioned as a protective factor against central obesity across both regions. However, PCW and CN modified such associations. In Yulin, PCW was associated with higher risk of obesity (OR = 1.11, 95% CI = 1.01, 1.23) and central obesity (OR = 1.31, 95% CI = 1.19, 1.44). In Shenzhen, PCW was associated with higher risk of obesity (OR = 1.23, 95% CI = 1.08, 1.39) and central obesity (OR = 1.61, 95% CI = 1.42, 1.82), all *p* < 0.001. The negative association between CN and obesity and central obesity were stronger in regional interaction, and with interaction coefficients of *β* = 1.37 and *β* = 1.28, respectively, both *p* < 0.001, as shown in Table 4.

## 4. Discussion

This study aimed to explore the relationship between parental feeding practices and children’s body composition. We explored associations between parental feeding practices and children’s body composition, building on evidence that these relationships may be bidirectional [24,25]. Although previous studies have investigated parental feeding behaviors, few have examined these relationships in the context of varying regional socioeconomic conditions. To address this gap, we analyzed data from two distinct regions in China, exploring how these associations vary according to regional socioeconomic status differences.

Firstly, our findings revealed significant gender differences in parental feeding practices. Parental occupation, education level, and household income are important factors influencing parental feeding decision-making. Notably, higher parental education levels were significantly associated with healthier body composition in children. Parents with higher educational attainment tend to prioritize dietary quality and nutritional balance, often providing healthier meals, which may contribute to lower body fat percentages in their children. These are key contributors to overweight and obesity in children, highlighting the need for parent interventions to improve feeding practices. Similarly, children and adolescents are in a critical stage of growth, undergoing significant changes in body composition, influenced by age and gender differences [26]. Chen et al. [27] reported significant differences in the percentile values of fat mass index among children and adolescents of different sexes and ages. Secondly, we observed that higher scores in PCW, CN, and PPW were associated with increased prevalence of overweight and obesity in children. Conversely, higher scores in PE were associated with decreased prevalence of these conditions. These findings suggest that appropriate parental feeding practices, including the application of PE when suitable, may contribute to reducing the rates of overweight, obesity, and central obesity in children. Interestingly, PE was a protective factor against central obesity. This finding suggests that, while parental encouragement to eat may promote healthy behaviors, it could also lead to unintended consequences, such as children’s resistance to food intake when they feel satiated. Thirdly, body composition metrics, including FMI and FFMI, were significantly correlated with PCW and CN across two regions in our research.

Furthermore, significant differences in children’s body composition were observed based on their place of residence. Children residing in Shenzhen, an economically developed city, exhibited healthier eating patterns, higher parental employment rates, and greater household income compared to children from less developed regions. These findings are consistent with previous studies, such as Baran et al.’s research [28] on urban and rural children in Poland, which demonstrated the influence of socio-environmental factors on body composition. Gender and place of residence emerged as crucial determinants of children’s body composition, underscoring the importance of context in addressing obesity in children [29]. Concern about child weight is a significant risk factor for obesity in children, independent of factors such as the child’s gender or parents’ educational background [30]. Research indicates that while concern about a child’s weight, dietary intake, and eating stress levels tend to increase, such this concern does not consistently translate into health-promoting parenting, potentially adversely harming children’s health [31]. Parental perceptions of their child’s weight status have been associated with an increased risk of overweight, obesity, and central obesity in children [32]. A study by Hidalgo-Mendez et al. [33] found that 97% of mothers of children with overweight or obesity underestimated their child’s weight status. This finding underscores a significant disconnect between parental perception and the child’s actual weight status. Furthermore, the child’s actual weight status was identified as the strongest predictor of maternal feeding practices, suggesting that accurate recognition of a child’s weight is essential for guiding appropriate parental interventions [34]. Additionally, PE, another critical factor affecting children with obesity, has been found to have a significant and negative association with both overweight and central obesity in children. This negative correlation was especially pronounced in Shenzhen, China, highlighting potential regional differences in feeding practices and their consequent effects on child health outcomes. Furthermore, a longitudinal study examining the relationship between PE and FMI and FFMI, higher PE pattern with lower FMI and FFMI [35]. This suggests that parental feeding stress may be significantly associated with emotional eating in children, such as reduced intake due to stress-induced negative emotions, potentially leading to lower body weight. These findings suggest that pressure to eat is linked to lower childhood weight, reinforcing the complexity of the relationship between psychological factors and obesity in children [36]. However, it needs to be understood in combination with behavioral and physiological mechanisms. Our study explores the relationship of different regions of parental feeding practices with their children’s body composition, and it suggests that perceived child weight and concern about child weight are stronger correlates of obesity and central obesity in children than other parental feeding practices. These associations were consistent across both regions studied, with particularly pronounced effects observed in Yulin, where higher rate of obesity and central obesity in children and the positive significance is more effective. If replicated in longitudinal studies, these findings may have several implications. First, promoting healthy parental feeding practice among parents in higher SES region. In contrast, for lower SES regions, alternative strategies are necessary, such as enhancing parental understanding of scientifically accurate perceptions of child weight and body shape to reduce unwarranted concerns about child weight. This study associates acculturation with favorable parental practices and their relationship with children’s body composition indicators, underscoring the importance of targeted education and awareness campaigns to improve parental feeding practices across different regions. Finally, the parental variables studied here explain a proportion of the variability in children’s nutritional health. It is imperative to integrate these findings with broader social, educational, and community determinants of child health, acknowledging that substantial work remains in these areas.

## 5. Strengths and Limitations

This study has several strengths. First, the sample was drawn from different cities, allowing for comparative analysis and enhancing the generalizability of the findings across diverse populations. Second, few studies have examined parental feeding practices in relation to children’s body composition. This gap makes it valuable to explore how parents—as primary caregivers—influence children’s growth patterns and body composition. Moreover, this study focuses on children’s body composition. whereas most existing research targets overweight or obesity in children. By accessing micro-level changes in body composition, we can better evaluate children’s nutritional health status. Relying solely on BMI may fail to accurately estimate fat content, potentially compromising research accuracy. Nevertheless, this study has limitations: the use of parent-reported questionnaires may introduce subjectivity and recall bias. Furthermore, as a cross-sectional design, it cannot establish causal relationships.

## 6. Conclusions

This study provides valuable insights into the association between parental feeding practices and children’s body composition and weight status across two distinct regions in China. Our findings revealed that child weight and concern about child weight were positively associated with body composition, while pressure to eat was negatively associated with a child’s body composition in two regions. In Shenzhen, parents are advised to adopt scientifically informed and accurate perceptions of their child’s weight and reduce excessive concern regarding weight status. While in Yulin, parents should improve parental awareness of healthy feeding practices and address potential misperceptions of child weight status. These results underscore the importance of considering regional and socioeconomic contexts when designing interventions to prevent and address obesity in children. Future research should further explore the mechanisms underlying these associations, particularly the role of cultural and socioeconomic factors in shaping parental feeding practices. Longitudinal studies are also warranted to establish causal relationships and evaluate the long-term effects of parental feeding practices on children’s health outcomes. By integrating these findings into public health policies may facilitate the development of tailored interventions to more effectively address obesity in children in different socioeconomic contexts.

## Figures and Tables

**Table 1:** Demographic characteristics of participants by sex and region (N = 1298).

	All	Sex				Region			
		Male (678)	Female (620)	t/x^2 a^	*p* ^a^	Yulin (650)	Shenzhen (648)	T/x^2 b^	*p* ^b^
Age(M ± SD)	10.65 ± 0.86	10.67 ± 0.03	10.62 ± 0.03	1.23	0.219	10.47 ± 0.05	10.82 ± 0.01	−7.30	<0.001
BMI (kg/m^2^)	18.99 ± 3.87	19.68 ± 4.07	18.24 ± 3.50	6.77	<0.001	19.24 ± 3.80	18.75 ± 3.94	2.32	0.021
WHtR	0.46 ± 0.06	0.48 ± 0.07	0.44 ± 0.05	11.03	<0.001	0.45 ± 0.06	0.46 ± 0.06	−3.17	0.002
Thin (%)	5.93	5.16	6.77	1.51	0.219	3.23	8.64	17.03	<0.001
Overweight (%)	15.64	19.47	11.45	15.78	<0.001	16.15	15.12	0.26	0.609
Obesity (%)	18.88	23.16	14.19	16.99	<0.001	21.23	16.51	4.72	0.030
Central obesity (%)	29.51	39.91	18.15	73.31	<0.001	27.38	31.67	2.85	0.091
Family characteristics									
Paternal profession (%)				6.57	0.161			61.85	<0.001
Agriculture, forestry, fishery and animal husbandry personnel	3.12	3.21	3.03			5.54	0.50		
Workers, staff, and staff members	34.43	35.11	33.67			33.38	35.56		
Responsible person and professional and technical personnel	13.85	15.73	11.78			8.62	19.53		
Soldiers and others	24.10	23.66	24.58			28.15	19.70		
People waiting for employment	24.50	22.29	26.94			24.31	24.71		
Maternal profession (%)				5.75	0.218			588.99	<0.001
Agriculture, forestry, fishery and animal husbandry personnel	1.20	1.68	0.67			1.38	1.00		
Workers, staff, and staff members	29.86	31.10	28, 28			12.46	48.75		
Responsible person and professional and technical personnel	14.89	15.57	14.14			9.85	20.37		
Soldiers and others	17.13	16.49	17.85			8.00	27.05		
People waiting for employment	36.91	34.96	39.06			68.31	28.40		
Paternal education (%)				0.32	0.850			444.65	<0.001
Middle school or lower	45.88	25.34	46.46			72.46	17.03		
High or vocational school	26.66	27.33	25.93			21.23	32.55		
College and above	27.46	27.33	27.61			6.31	50.42		
Maternal education (%)				1.02	0.599			460.47	<0.001
Middle school or lower	43.72	42.60	44.95			71.08	14.02		
High or vocational school	26.98	28.09	25.76			20.92	33.56		
College and above	29.30	29.31	29.29			8.00	52.42		
Income (Yuan)				1.06	0.303			693.34	<0.001
<10,000	59.33	58.63	60.10			94.46	21.20		
≥10,000	40.67	41.37	39.90			5.54	78.80		
Maternal BMI (kg/m^2^)	22.26 ± 4.59	23.81 ± 0.15	24.18 ± 0.19	−1.52	0.130	23.93 ± 0.21	24.05 ± 0.12	−0.52	0.605
Paternal BMI (kg/m^2^)	23.99 ± 4.38	22.18 ± 0.17	22.35 ± 0.19	−0.68	0.497	22.61 ± 0.21	21.91 ± 0.14	2.73	0.006
SES ^c^				2.29	0.318			191.83	<0.001
Low	38.83	37.71	40.07			70.46	4.51		
Middle	32.91	33.89	31.82			27.54	38.73		
High	28.26	28.40	28.11			13.00	56.76		
Parental feeding practices (M ± SD)									
Monitoring	13.73 ± 4.18	13.73 ± 0.15	13.73 ± 0.17	−0.01	0.998	13.14 ± 0.18	14.32 ± 0.14	−5.16	<0.001
Pressure to Eat	12.63 ± 3.56	12.65 ± 0.13	12.60 ± 0.15	0.22	0.824	12.81 ± 0.15	12.45 ± 0.13	1.83	0.068
Restriction	22.08 ± 5.77	22.11 ± 0.22	22.06 ± 0.24	0.15	0.881	21.70 ± 0.25	22.48 ± 0.20	−2.44	0.015
Perceived Child Weight	11.61 ± 2.16	11.77 ± 0.08	11.43 ± 0.09	2.85	0.004	11.44 ± 0.09	11.78 ± 0.08	−2.83	0.005
Concern about Child Weight	6.52 ± 3.27	6.62 ± 0.13	6.41 ± 0.13	1.15	0.249	7.63 ± 0.13	5.40 ± 0.11	13.07	<0.001
Perceived Parent Weight	8.52 ± 1.76	8.51 ± 0.07	8.54 ± 0.07	−0.27	0.791	8.73 ± 0.07	8.31 ± 0.07	4.29	<0.001
Food as Reward	6.16 ± 2.30	6.29 ± 0.09	6.02 ± 0.09	2.13	0.033	6.11 ± 0.09	6.22 ± 0.09	−0.83	0.409

^a^ Male vs. Female. ^b^ Shenzhen vs. Yulin. ^c^ SES (socioeconomic status): a continuous composite index of socioeconomic status (SES) was constructed using principal component analysis (PCA) based on five variables: the educational level, occupation and monthly family income of fathers and mothers. The resulting SES scores were categorized into tertiles (low, medium, high) using the third quartile method, providing a comprehensive reflection of relative socioeconomic status levels.

**Table 2:** Descriptive characteristics of parental feeding practices and body composition in two regions. [N (%)/M (SD)].

	MN		PE		RST		PCW		CN		PPW		FR	
	Lower	Higher	Lower	Higher	Lower	Higher	Lower	Higher	Lower	Higher	Lower	Higher	Lower	Higher
**Yulin**														
Overweight and Obesity (%)	140 (38.90)	94 (35.21)	155 (42.23)	88 (31.10) *	116 (35.47)	124 (39.32)	154 (29.28)	89 (71.77) **	80 (28.88)	163 (43.70) **	184(34.20)	59 (52.68) **	154 (43.02)	89 (30.48) *
Central obesity (%)	105 (27.42)	73 (27.34)	117 (31.88)	61 (21.55) *	86 (26.30)	92 (28.48)	103 (19.58)	75 (60.48) **	55 (19.86)	123 (32.98) **	129(23.98)	49 (43.75) **	112 (31.28)	66 (22.60) *
FMI (kg/m)^2^	5.60 ± 2.61	5.73 ± 2.89	5.98 ± 2.92	5.23 ± 2.39 **	5.46 ± 2.55	5.85 ± 2.89	5.10 ± 2.31	7.99 ± 3.09 **	5.02 ± 2.34	6.12 ± 2.90 **	4.70 ± 2.88	6.15 ± 3.47 **	5.84 ± 2.67	5.43 ± 2.78
FFMI (kg/m)^2^	13.45 ± 1.43	13.42 ± 1.45	13.56 ± 1.50	13.27 ± 1.34 *	13.42 ± 1.38	13.44 ± 1.50	13.22 ± 1.31	14.35 ± 1.58 **	13.16 ± 1.37	13.63 ± 1.46 **	13.88 ± 1.39	14.48 ± 1.45 **	13.56 ± 1.42	13.28 ± 1.45 *
**Shenzhen**														
Overweight and Obesity (%)	91 (30.23)	114 (32.85)	152 (38.00)	53 (21.37) **	99 (30.46)	106 (32.82)	78 (17.26)	127 (64.8) **	88 (19.09)	117 (62.90) **	161(29.38)	44 (44.44) *	99 (31.83)	106 (31.55)
Central obesity (%)	90 (30.30)	113 (32.85)	152 (38.48)	51 (20.73) **	97 (30.12)	106 (33.23)	91 (20.31)	112 (58.03) **	96 (32.98)	107 (58.79) **	159(29.28)	44 (45.36) *	95 (31.05)	108 (32.34)
FMI (kg/m2)	4.15 ± 3.19	4.27 ± 3.10	4.78 ± 3.41	3.31 ± 2.39 **	4.07 ± 2.83	4.36 ± 3.41	3.19 ± 2.12	6.58 ± 3.78 **	3.23 ± 2.06	6.65 ± 3.94 **	4.70 ± 2.88	6.15 ± 3.47 **	4.31 ± 3.17	4.13 ± 3.12
FFMI (kg/m2)	14.49 ± 1.22	14.56 ± 1.11	14.70 ± 1.09	14.24 ± 1.23 **	14.54 ± 1.10	14.51 ± 1.23	14.23 ± 1.06	15.21 ± 1.10 **	14.27 ± 1.08	15.14 ± 1.13 **	13.88 ± 1.39	14.48 ± 1.45 **	14.56 ± 1.12	14.49 ± 1.21

Abbreviations: FMI: Fat mass index; FFMI: Fat free mass index. * *p* < 0.05, ** *p* < 0.001.

**Table 3:** Multivariable regression analysis for body composition on CFQ factors.

		FMI (kg/m^2^) [β(95% CI)]	FFMI (kg/m^2^) [β(95% CI)]
MN	Yulin	0.02 (−0.02, 0.07)	0.01 (−0.02, 0.03)
	Shenzhen	0.05 (−0.01, 0.12)	0.02 (−0.01, 0.04)
	Region * MN	0.02 (−0.07, 0.10)	0.00 (−0.03, 0.04)
PE	Yulin	−0.11 (−0.17, −0.06) **	−0.04 (−0.06, −0.01) *
	Shenzhen	−0.25 (−0.32, −0.18) **	−0.06 (−0.08, −0.03) **
	Region * PE	−0.14 (−0.22, −0.05) *	−0.02 (−0.06, 0.02)
RST	Yulin	0.03 (−0.01, 0.06)	0.01 (−0.003, 0.03)
	Shenzhen	0.03 (−0.02, 0.08)	0.00 (−0.02, 0.02)
	Region * RST	−0.00 (−0.06, 0.06)	−0.01 (−0.04, 0.01)
PCW	Yulin	0.39 (0.30, 0.47) **	0.17 (0.13, 0.22) **
	Shenzhen	0.67 (0.56, 0.78) **	0.22 (0.18, 0.26) **
	Region * PCW	0.32 (0.18, 0.46) **	0.06 (−0.00, 0.11)
CN	Yulin	0.17 (0.11, 0.23) **	0.07 (0.04, 0.10) **
	Shenzhen	0.56 (0.48, 0.64) **	0.12 (0.09, 0.16) **
	Region * CN	0.44 (0.34, 0.54) **	0.07 (0.02, 0.11) *
PPW	Yulin	0.25 (0.13, 0.37) **	0.10 (0.04, 0.16) *
	Shenzhen	0.15 (0.01, 0.29) *	0.12 (0.07, 0.17) **
	Region * PPW	−0.05 (−0.22, 0.14)	0.03 (−0.05, 0.10)
FR	Yulin	−0.08 (−0.17, 0.01)	−0.06 (−0.10, −0.02)
	Shenzhen	−0.09 (−0.19, 0.02)	−0.02 (−0.05, 0.02)
	Region * FR	0.01 (−0.13, 0.15)	0.04 (−0.01, 0.10)

Abbreviations: FMI: Fat mass index; FFMI: Fat free mass index. MN: Monitoring; PE: Pressure to eat; RST: Restriction; PCW: Perceived child weight; CN: Concern about child weight; PPW: Perceived parent weight; FR: Food as reward. ** *p* < 0.001; * *p* < 0.01. All models adjusted for child’s age, sex, maternal and parental education, maternal and paternal profession, family monthly income, maternal and paternal BMI.

**Table 4:** Multivariable regression analysis between CFQ factors and child weight status.

		Obesity [OR (95%CI)]	Central Obesity [OR (95%CI)]
MN	Yulin	0.97 (0.92, 1.02)	1.00 (0.96, 1.04)
	Shenzhen	1.01 (0.94, 1.08)	1.04 (0.99, 1.10)
	Region × MN	1.03 (0.95, 1.11)	1.03 (0.96, 1.10)
PE	Yulin	1.00 (0.94, 1.06)	0.92 (0.87, 0.96) *
	Shenzhen	0.95 (0.89, 1.02)	0.86 (0.81, 0.92) **
	Region × PE	0.93 (0.85, 1.02)	0.96 (0.88, 1.03)
RST	Yulin	1.01(0.98, 1.05)	1.00 (0.97, 1.03)
	Shenzhen	0.99 (0.95, 1.04)	1.02 (0.98, 1.06)
	Region × RST	1.01(0.95, 1.06)	1.01 (0.96, 1.06)
PCW	Yulin	1.11 (1.01, 1.23) **	1.31 (1.19, 1.44) **
	Shenzhen	1.23 (1.08, 1.39) **	1.61 (1.42, 1.82) **
	Region × PCW	1.34 (1.12, 1.61) *	1.18 (1.02, 1.38) *
CN	Yulin	1.04 (0.98, 1.11)	1.12 (1.07, 1.19) **
	Shenzhen	1.08 (0.99, 1.17)	1.45 (1.32, 1.58) **
	Region × CN	1.37 (1.22, 1.53) **	1.28 (1.16, 1.41) **
PPW	Yulin	1.07 (0.95, 1.21)	1.15 (1.03, 1.28)
	Shenzhen	1.05 (0.91, 1.20)	1.13 (1.01, 1.27)
	Region × PPW	0.99(0.83, 1.18)	0.99(0.85, 1.16)
FR	Yulin	0.93(0.85, 1.02)	0.93(0.86, 1.00)
	Shenzhen	0.97(0.87, 1.07)	0.97(0.89, 1.06)
	Region × FR	1.08(0.95, 1.23)	1.07(0.95, 1.19)

Abbreviations: MN: Monitoring; PE: Pressure to eat; RST: Restriction; PCW: Perceived child weight; CN: Concern about child weight; PPW: Perceived parent weight; FR: Food as reward. ** *p* < 0.001; * *p* < 0.01. All models adjusted for child’s age, sex, maternal and paternal education, maternal and paternal profession, family monthly income, maternal and paternal BMI.

## Data Availability

The data can be retrieved from the corresponding author upon reasonable request.

## Abbreviations

The following abbreviations are used in this manuscript:

BMI	Body mass index
FMI	Fat mass index
FFMI	Fat free mass index
WHtR	Waist to height ratio
MN	Monitoring
PE	Pressure to eat
RST	Restriction
PCW	Perceived child weight
CN	Concern about child weight
PPW	Perceived parent weight
FR	Food as reward
